# Phosphorylation of the Actin Binding Protein Drebrin at S647 Is Regulated by Neuronal Activity and PTEN

**DOI:** 10.1371/journal.pone.0071957

**Published:** 2013-08-05

**Authors:** Patricia Kreis, Rita Hendricusdottir, Louise Kay, Ismini E. Papageorgiou, Michiel van Diepen, Till Mack, Jonny Ryves, Adrian Harwood, Nicholas R. Leslie, Oliver Kann, Maddy Parsons, Britta J. Eickholt

**Affiliations:** 1 MRC Centre for Developmental Neurobiology, King’s College London, London, United Kingdom; 2 Department of Pharmacology, University of Oxford, Oxford, United Kingdom; 3 Institute of Physiology and Pathophysiology, University of Heidelberg, Heidelberg, Germany; 4 Novartis Pharmaceuticals UK Limited, Horsham, United Kingdom; 5 Cluster of Excellence NeuroCure and Institute of Biochemistry, Charité - Universitätsmedizin Berlin, Berlin, Germany; 6 Cardiff School of Biosciences, Cardiff University, Cardiff, United Kingdom; 7 College of Life Sciences, University of Dundee, Dundee, United Kingdom; 8 The Randall Division of Cell and Molecular Biophysics, King’s College London, London, United Kingdom; Virginia Tech Carilion Research Institute, United States of America

## Abstract

Defects in actin dynamics affect activity-dependent modulation of synaptic transmission and neuronal plasticity, and can cause cognitive impairment. A salient candidate actin-binding protein linking synaptic dysfunction to cognitive deficits is Drebrin (DBN). However, the specific mode of how DBN is regulated at the central synapse is largely unknown. In this study we identify and characterize the interaction of the PTEN tumor suppressor with DBN. Our results demonstrate that PTEN binds DBN and that this interaction results in the dephosphorylation of a site present in the DBN C-terminus - serine 647. PTEN and pS647-DBN segregate into distinct and complimentary compartments in neurons, supporting the idea that PTEN negatively regulates DBN phosphorylation at this site. We further demonstrate that neuronal activity increases phosphorylation of DBN at S647 in hippocampal neurons *in vitro* and in *ex vivo* hippocampus slices exhibiting seizure activity, potentially by inducing rapid dissociation of the PTEN:DBN complex. Our results identify a novel mechanism by which PTEN is required to maintain DBN phosphorylation at dynamic range and signifies an unusual regulation of an actin-binding protein linked to cognitive decline and degenerative conditions at the CNS synapse.

## Introduction

PTEN (Phosphatase and tensin homolog) was originally identified as a tumor suppressor that negatively regulates the Phosphatidylinositol 3-kinase (PI3K) signaling pathway [1]. Human germline PTEN mutations or conditional deletions of PTEN in mice have further been associated with neurological disorders such as macrocephaly, seizures, mental retardation and autism [2–6]. Neuronal deficiencies leads to several abnormal morphological features, including neuron hypertrophy, ectopic dendrites, aberrant axonal projections and increased dendritic spine density, as well as aberrant neuronal transmission [5,7]. Whilst most of the characterized neuronal responses can be credited to PTEN’s role in the regulation PI3K signaling [8–10], PTEN has other potential mechanisms of action including functions independent of the lipid phosphatase activity and functions in the nucleus [11,12]. The physiological significances of these PI3K-independent roles, especially in neurons, remain largely unclear.

In order to understand the spatial and temporal regulation of PTEN function in the brain, we searched for new PTEN protein–protein interactions using mass spectrometry. Our search identified a new binding partner: Drebrin (developmentally regulated brain protein, DBN), a protein that binds to actin filaments. In adult neurons, DBN accumulates in regions highly enriched in F-actin, such as neuronal growth cones and dendritic spines, and modulates synaptic plasticity by affecting the spine morphology and by regulating neuronal transmission [13,14]. Localization of DBN is important for the function of DBN in postsynaptic regulation, and there is evidence that clustering of DBN in dendritic spines is regulated by AMPA (2-amino-3-(3-hydroxy-5-methyl-isoxazol-4-yl)propanoic acid) receptor activity [15]. DBN also associates with several important postsynaptic signaling proteins; for example, it regulates the synaptic targeting of NMDA (N-Methyl-D-aspartate) receptors [16], it interacts with the scaffolding protein Homer [17] and it induces the accumulation of PSD95 (Postsynaptic density protein 95) in dendritic spines [18]. Interestingly, reduced levels of DBN have been observed in the hippocampus of patients with Alzheimer’s disease [19].

We show here that PTEN interacts directly with DBN and negatively regulates levels of S647-phosphorylation of DBN independently of PI3K. Neuronal activity induces a dissociation of the PTEN:DBN complex and de-represses S647-DBN phosphorylation leading to an increase in S647-phosphorylation. Our findings provide new molecular insights into how PTEN may control synaptic functions by targeting the actin binding protein DBN.

## Results

**Figure 1:**
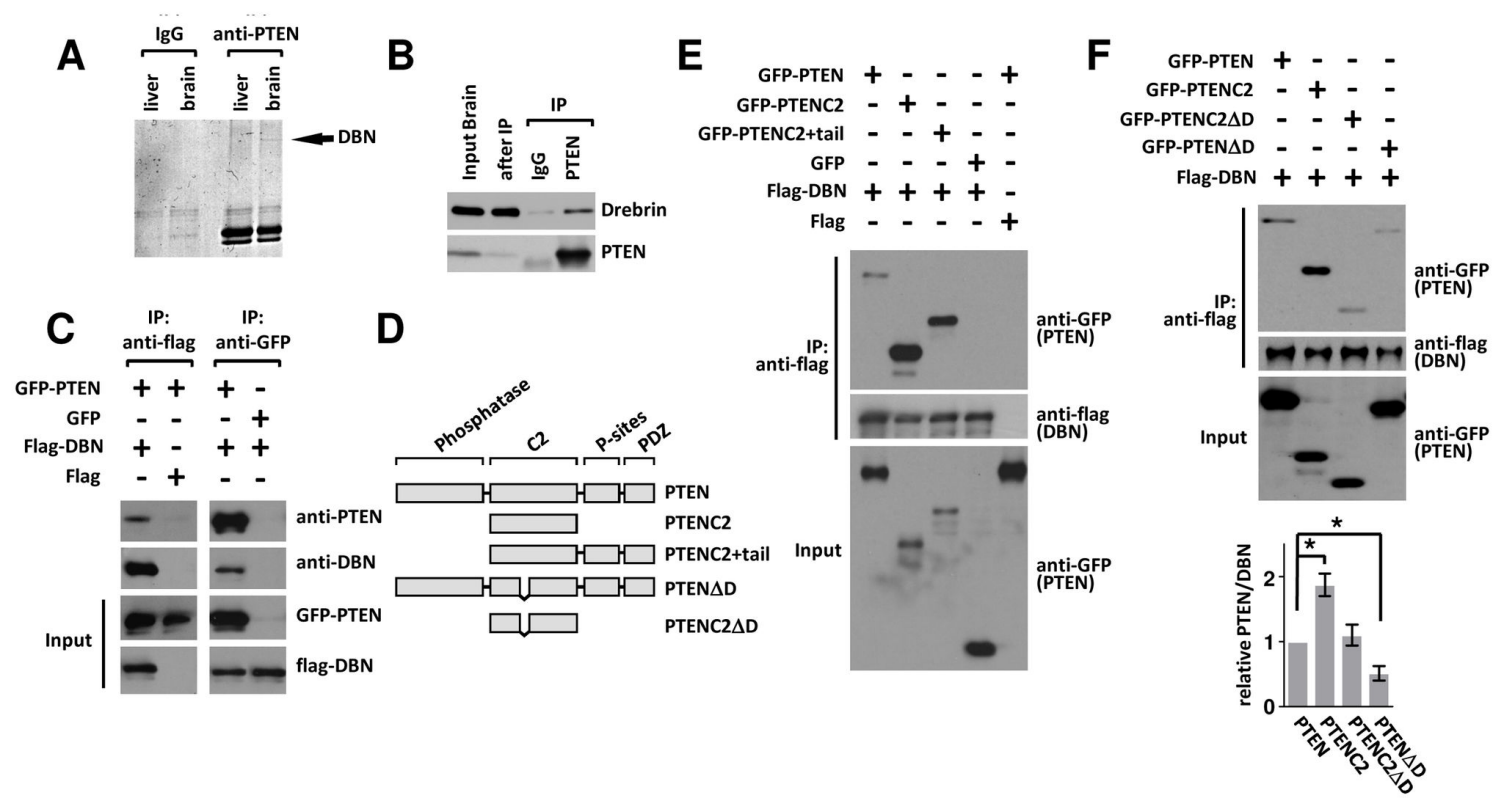
The PTEN-DBN interaction requires an intact PTEN D-loop. (A) Coomassie blue stained gel of immunoprecipitated PTEN from E18 rat brain and liver. The arrow indicates the band excised from the gel for mass spectrometry analysis, which was identified as drebrin (DBN). (B) E18 rat brain lysate was incubated with anti-PTEN antibody or control IgG; IPs were analyzed with indicated antibodies. (C) GFP-PTEN (or control GFP) and Flag-DBN (or control Flag) were co-expressed in HEK293 cells. Following anti-flag (left) or anti-GFP (right) IP, co-precipitates were analyzed with anti-PTEN and anti-DBN antibodies. (D) Domain structure of PTEN and PTEN-deletion constructs. PTEN consists of an N-terminal phosphatase domain that can act on both protein and lipid substrates. The C-terminal domain contains a C2 domain and a cluster of phosphorylation sites. In the extreme C-terminus of PTEN a binding motif for PDZ domains is present. (E, F) HEK293 cells were co-transfected with indicated constructs before immunoprecipitation as described in (C). Blots were analyzed with indicated antibodies. (E) In comparison to full-length PTEN, the PTENC2 domain shows increased binding to DBN. (F) The PTEN: DBN interaction requires an intact D-loop (aa 281-312). Bar graph represents the average band density of GFP-PTEN/ FLAG-DBN in the co-IP in three different experiments + sem. *p<0.05 relative to wt PTEN.

We performed mass spectrometry analysis of PTEN complexes from liver and brain and identified a brain-specific PTEN interaction of approximately 110 kD, Drebrin (DBN) (Figure 1A); 5 peptides matched the DBN entry (Q07266), with a total coverage of 15%.

### The PTEN-DBN interaction requires an intact PTEN D-loop

DBN is an actin-binding protein that accumulates in regions enriched in F-actin, such as dendritic spines, and modulates synaptic plasticity by affecting spine morphology and by regulating neuronal transmission [13,14]. Initial characterization verified the PTEN-DBN interaction by co-immunoprecipitation (co-IP) from rat brain lysate (Figure 1B). To confirm the interaction, FLAG-DBN and GFP-PTEN were transiently expressed in HEK293 cells, and Flag-DBN (or GFP-PTEN) protein complexes immunoprecipitated using anti-Flag-M2 (or anti-GFP antibodies). Western blot analysis using an anti-PTEN (or anti-DBN) antibody identified the immunoprecipitated protein complexes (Figure 1C). In order to further characterize the interactions we coexpressed FLAG-DBN with different GFP-PTEN deletions (Figure 1D–F). These experiments identified that in comparison to wildtype PTEN, a PTEN fragment encompassing only the structurally defined C2 domain (amino-acid 182-354; PTEN C2) greatly increased the interaction with DBN (Figure 1E) and suggested that the PTEN:DBN interaction may be mediated through the PTEN C2 domain. The PTEN C2-domain incorporates a stretch of highly conserved amino acids, the ‘deleted-loop’ (D-loop; aa 286-309), which is susceptible to proteolysis [20] and subject to posttranslational modification by mono- and poly-ubiquination [21]. We found that deletions of the D-loop from full length PTEN (PTENΔD) and from the PTEN C2-domain (PTENC2ΔD) decreased the DBN interaction in comparison to the parent proteins PTEN and PTENC2, respectively (Figure 1F).

**Figure 2:**
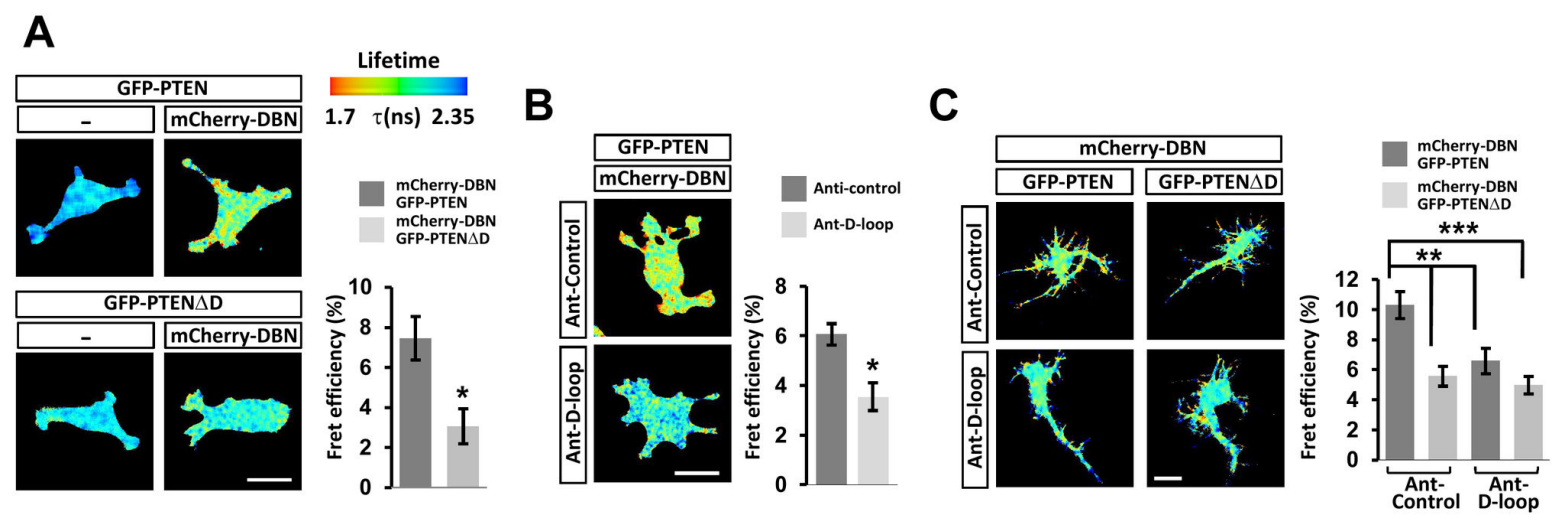
Analysis of the PTEN:DBN interaction by FRET in PC12 cells using multiphoton FLIM. (A) Images show the lifetime maps of FRET across cells using a pseudocolour scale (blue, normal GFP lifetime; red, FRET). GFP-PTEN (**top left**) and GFP-PTENΔD (**bottom left**) demonstrate normal GFP lifetime in the absence of acceptor, other images show co-expression of GFP-PTEN (or GFP-PTENΔD) with mCherry-DBN. Bar graph representing the average FRET efficiency of 16 cells over 3 independent experiments + sem. *p<0.01. (B) Cells were incubated for 1 hour with scrambled control peptide (control) or a small peptide derived from the PTENC2 domain (D-loop), before FRET analyses. Both peptides are fused to the antennapedia (Ant) internalisation sequence. Bar graphs represent the average FRET efficiency of 13 cells over 3 independent experiments + sem. *p<0.01. (C) Hippocampal neurons were transfected at 7DIV and incubated at 10DIV with Ant-control or Ant-D-loop peptides. Bar graphs represent the average FRET efficiency of 14 cells over 3 independent experiments + sem. **p<0.005, ***p<0.001. Scale bars: 10 µm.

We further analyzed the interaction by measuring fluorescence resonance energy transfer (FRET) using multiphoton fluorescence-lifetime imaging microscopy (FLIM). Expression of GFP-PTEN or GFP-PTENΔD alone demonstrated normal GFP lifetime in the absence of acceptor, whilst co-expression of GFP-PTEN with mCherry-DBN induced a GFP lifetime reduction, indicative of direct protein–protein interaction (Figure 2A). When compared to the GFP-PTEN and mCherry-DBN pair, and in agreement with our co-IP experiments, we found significantly reduced FRET efficiencies when we measured lifetime reductions between GFP-PTENΔD and DBN (Figure 2A), or between GFP-PTEN and mCherry-DBN in the presence of cell-permeant peptide encompassing the D-loop sequence fused to Antennapedia [22] (Figure 2B). Similar results were obtained in neurons: When compared to GFP-PTEN and mCherry-DBN, FRET signals were decreased between the GFP-PTENΔD deletion mutant and mCherry-DBN. Presence of the Ant-D-loop peptide did not result in further FRET reductions (Figure 2C), indicating that the peptide can be used specifically to reduce binding of PTEN to DBN by competing for PTEN D-loop interactions.

**Figure 3:**
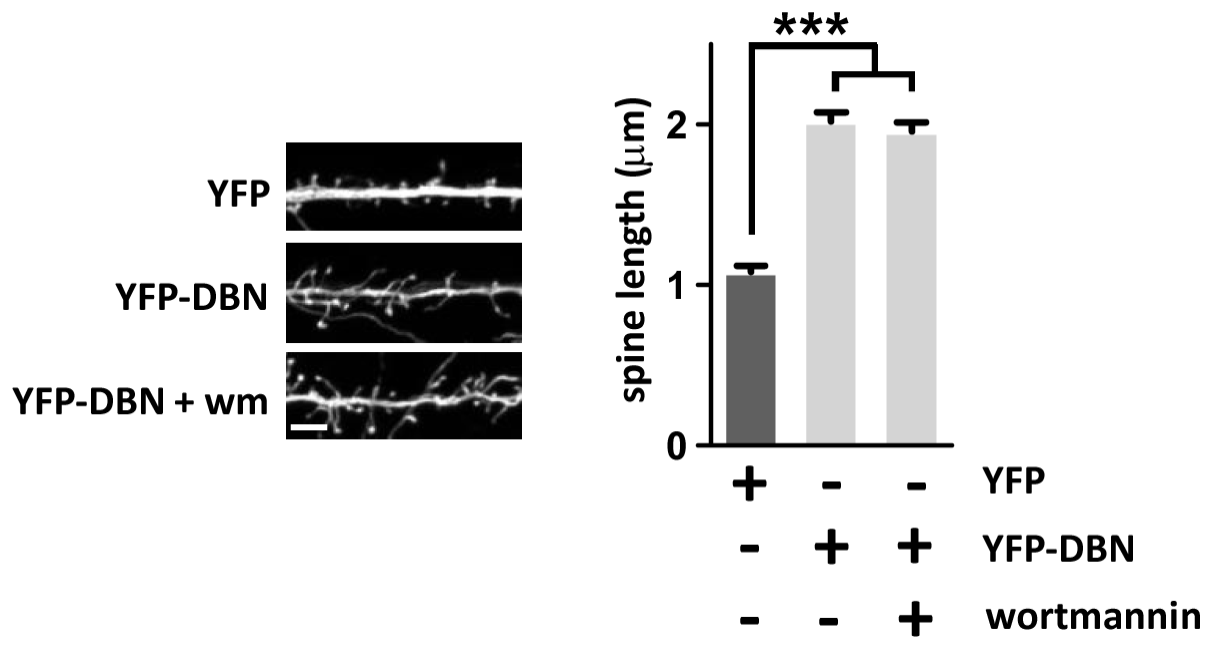
Drebrin induced dendritic protrusions do not require PI3K. Rat hippocampal neurons were transfected at 7 DIV with YFP or YFP-DBN. The PI3K inhibitor wortmannin was applied at 100 nM following transfection. At 17 DIV, neurons were fixed and the length of individual dendrite protrusions was determined from the base to the tip of the spine head using Neuron-J. To avoid spine variability, we restricted the analysis to the primary and secondary dendrites 100-150 µm away from the soma. Each data point represent the relative length of at least 220 protrusions measured over 3 independent experiments + sem. ***p<0.001. Scale bar: 3 µm.

We surmised that the association of PTEN with the actin binding protein DBN may govern essential processes during the re-organization of the actin cytoskeleton at the synapse, potentially involving PI3K dependent signaling mechanisms. Both expression of DBN and overactivation of PI3K signaling through overexpression of constitutively active PI3K p110 subunits induce longer dendritic spines [7,23,24] suggesting an interaction of PI3K/PTEN signaling and DBN during dendrite filopodia or synapse formation. However, application of pharmacological inhibition of PI3K by Wortmannin did not modify the increase in spine length induced by DBN overexpression (Figure 3).

### Drebrin is phosphorylated at S647

**Figure 4:**
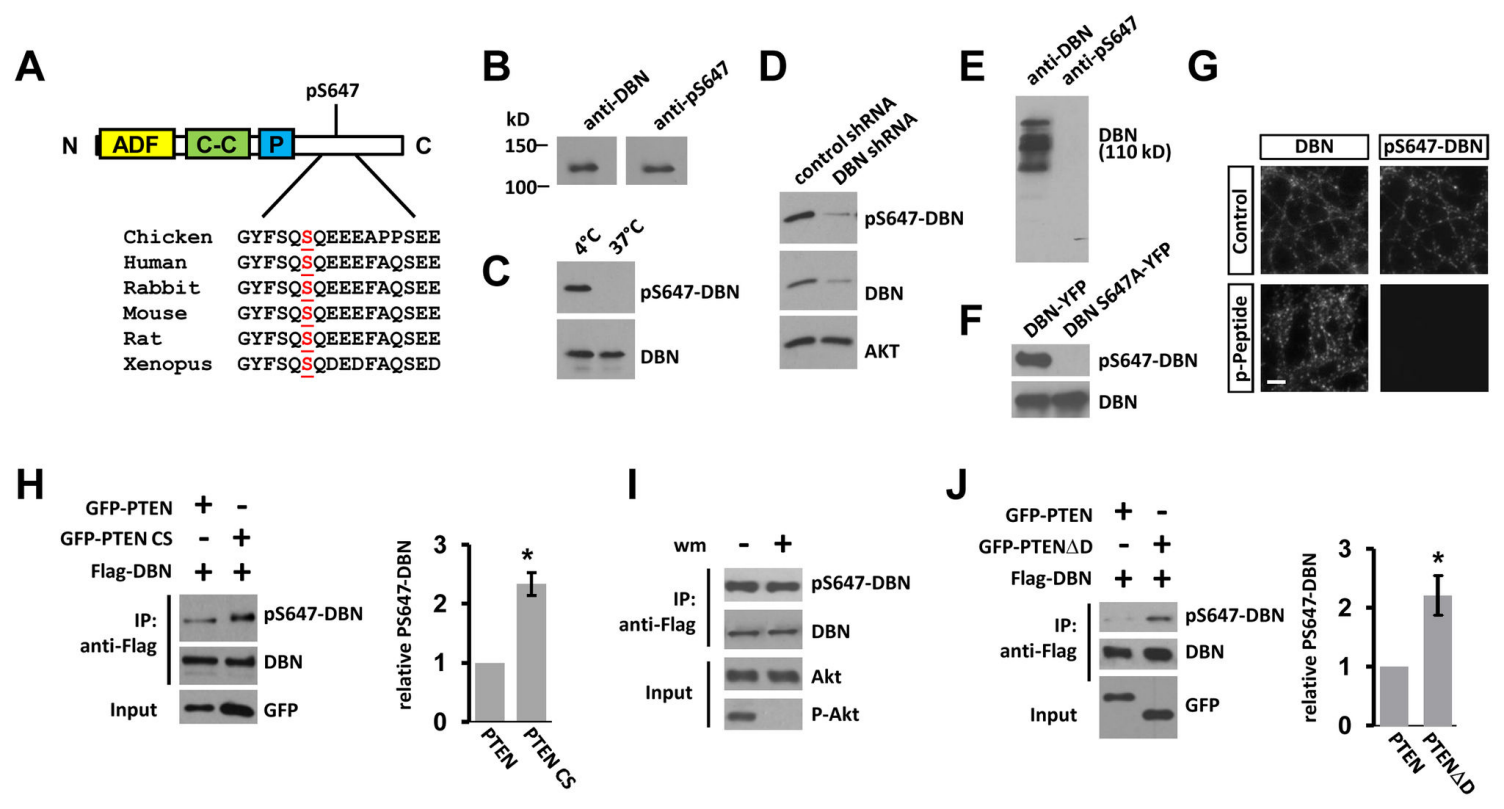
pS647-Drebrin is de-phosphorylated in dependence of the PTEN phosphatase activity, but independent of PI3K signalling. (A) Alignment of S647 DBN. DBN comprises of an N-terminal ADF/cofilin homology domain (ADF), a coiled-coil (C–C) domain, a proline-rich stretch (P) and an unstructured C-terminus. (B) Adult rat brain homogenate was analysed by western blot analysis and probed with anti-DBN antibody or with the affinity purified pS647-DBN antibody. (C) Brain homogenate was incubated on ice or at 37°C for 1 hour to activate endogenous phosphatases, before western blotting and analyses. (D) Cortical neurons were nucleofected with control or DBN specific shRNA. Neuronal lysate was analysed at 3 DIV. (E) Bacterially expressed and purified His-DBN was probed for anti-DBN and anti-pS647-DBN antibodies. (F) Cell lysates prepared from YFP-DBN or YFP-S647A-DBN expressing HEK293 cells were analysed by western blotting using indicated antibodies. (G) 19 DIV hippocampal neurons were stained with pan-drebin (mouse) and pS647-DBN antibodies (rabbit) in the presence of control serum or with the pS647-DBN peptide. Scale bar: 10 µm. (H) GFP-PTEN or the GFP-PTEN CS mutant was co-expressed with Flag-DBN in U87MG cells. Bar graph represents the average band density of pS647-DBN/DBN in 3 independent experiments + sem. *p<0.05. (I) Flag-DBN expressing U87MG cells were treated with wortmannin (wm) for 1 hour at 100 nM. (J) As in (H), but cells were co-transfected with Flag-DBN and GFP-PTEN, or with Flag-DBN and GFP-PTENΔD. Bar graph (right) represents the average band density of pS647-DBN/DBN in 3 independent experiments + sem. *p<0.05.

In search for potential effects of PTEN’s association with DBN, we considered the presence of DBN amino acid residues that could be phosphorylated in dependence of PTEN. Indeed, DBN has been shown to be highly phosphorylated at a number of residues in different tissues, including neuronal tissue [25–34]. We focused on a phosphorylation site embedded in a highly conserved region within the DBN C-terminus - serine 647 (S647) - and raised an anti-DBN-S647 phospho-site-specific antibody (Figure 4A,B). Following affinity purification we found that the antibody detects a band of approximately 110kD in brain lysates, but not in lysates in which endogenous phosphatases were activated by incubation at 37^o^C (Figure 4C). Similarly, in cell extracts obtained from cortical neurons expressing DBN specific siRNA, the detection of DBN as well as the S647 phosphorylated form of DBN, was greatly decreased (Figure 4D). The antibody also failed to detect bacterially expressed (phosphorylation-deficient) DBN and a mutant version of YFP-DBN in which S647 was replaced with an alanine (S647A) (Figure 4E, F). The specificity of pS647-DBN antibody was also demonstrated by incubating anti-pS647-DBN with the pS647-DBN peptide prior to staining of hippocampal neurons cultured *in vitro* (Figure 4G). Together, these findings indicate that anti-pS647-DBN specifically recognizes the S647-phosphorylated form of DBN, and that this phosphorylation event readily occurs in brain tissue and in hippocampal neuronal cultures.

### PTEN negatively regulates S647-phosphorylation of DBN independent of PI3K

We next sought to gain insight into a potential regulation of pS647-DBN by PTEN, and co-expressed flag-tagged DBN in PTEN-deficient U87MG cells with PTEN or with a phosphatase-dead PTEN mutant (PTEN C124S). Analyses of FLAG IPs indicated that pS647-DBN levels were significantly reduced when a phosphatase active PTEN was present (Figure 4H). PTEN is thought to function primarily as an antagonist of PI3K signaling; therefore, we tested if direct inhibition of PI3Ks using pharmacological approaches affected levels of S647-DBN phosphorylation. Interestingly, treatment of the cells with wortmannin did not alter pS647-DBN, indicating that pS647-DBN is sensitive to a PTEN signaling pathway that operates independently of PI3K (Figure 4I). We then compared the efficiency of PTEN in regulating pS647-DBN also with that of PTENΔD, which demonstrated reduced interaction with DBN (Figure 1). Again, wild type PTEN significantly reduced phosphorylation of DBN at S647 when compared to PTENΔD (Figure 4J). Because the D-loop-deficient PTENΔD mutant retains wild-type PTEN catalytic activity [20], this result indicates that an intact PTEN:DBN association was crucial for the PTEN effect towards pS647-DBN.

### S647-phosphorylation of DBN and PTEN segregate into opposite neuronal compartments

**Figure 5:**
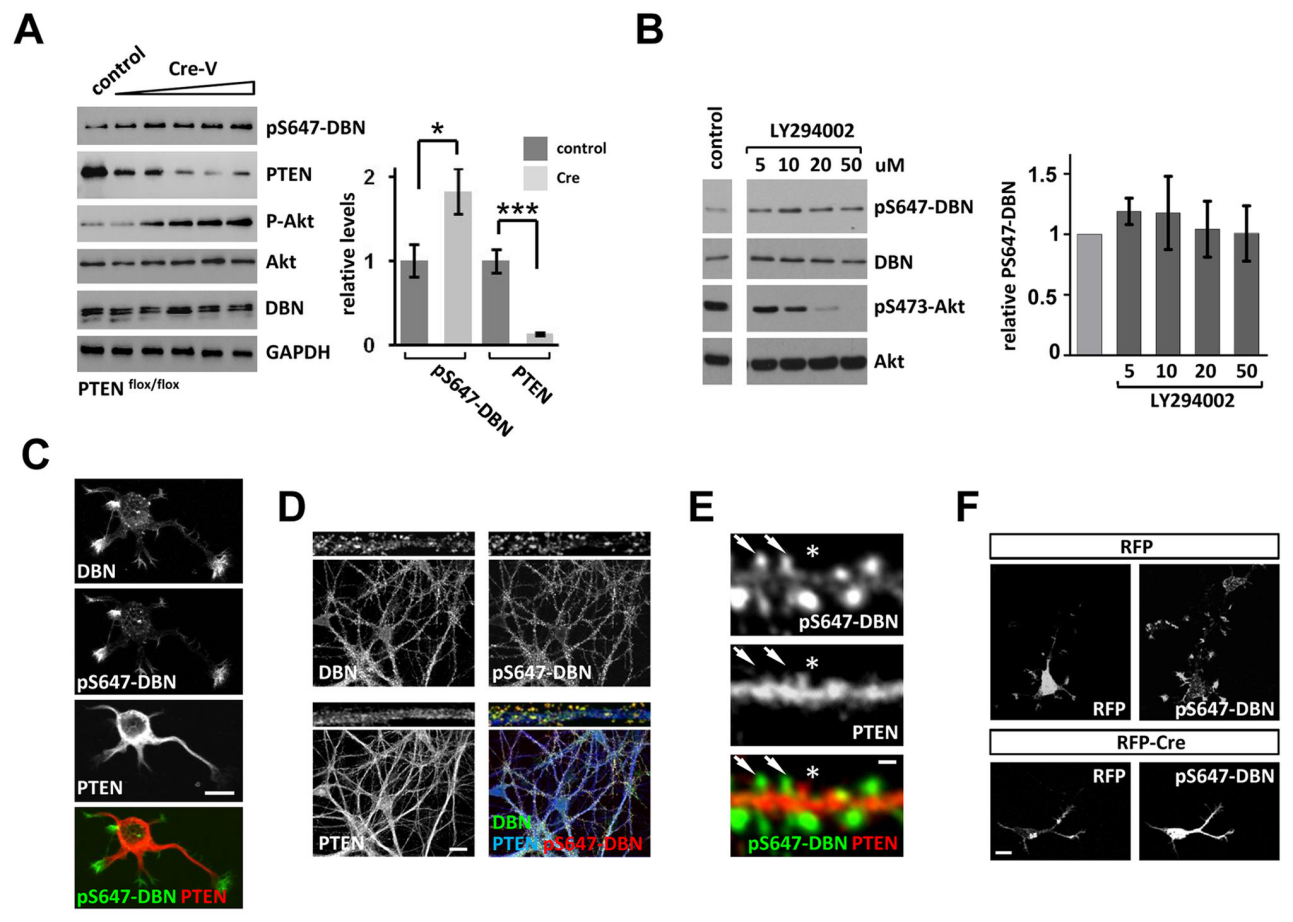
pS647-Drebrin is negatively regulated by PTEN in neurons independently of PI3K. (A) Cortical neurons isolated from *PTEN* ^*flox/flox*^ mice were transduced with increasing concentrations of Cre-expressing viruses at 13 DIV. Neuronal cell lysates were analysed at 19 DIV. Bar graph represents the relative band density of pS647-DBN and PTEN in three independent experiments with maximal cre-virus titer used, + sem. *p<0.05, *** < 0.001. (B) Rat cortical neurons were treated with the PI3K inhibitor LY294002 at indicated concentrations for 60 minutes before analyses of neuronal cell lysates. Bar graph represents the average band density of pS647-DBN in three independent experiments. (C) Hippocampal neurons were cultured for 1.5 DIV and labelled with anti-DBN, anti-pS647-DBN and anti-PTEN antibodies. (D) 18 DIV hippocampal neurons were stained with anti-DBN, anti-pS647-DBN and anti-PTEN. Top images show a specimen images at higher magnification. DBN is highly enriched in dendritic spines, but also found - albeit at lower levels - in the dendrite and the soma, whereas phosphorylation at S657 is more confined to the dendritic spine compartment. (E) Hippocampal neurons were cultured for 18 DIV and stained with anti-pS647-DBN and anti-PTEN antibodies. In dendritic spines (**arrows**), DBN is highly phosphorylated on pS647, whereas PTEN is mainly present in the dendrite process. Sporadically, PTEN is found in spines (**star**), which coincides with depleted pS647-Drebrin labeling. (F) Cortical neurons isolated from PTEN floxed/floxed mice were nucleofected with RFP or Cre-RFP and cultured for DIV3 before staining for pS647-DBN. Scale bars C, D, F: 10 µm; E: 2 µm.

Next, we tested for the existence of similar PTEN signaling mechanisms in neurons and removed PTEN in neurons obtained from *PTEN* ^*flox/flox*^ mice through Cre-mediated recombination. Infection of cortical neuronal cultures with Cre expressing viruses caused a reduction in PTEN protein levels to approximately 10%, resulting in a rise in Akt phosphorylation that coincided with increased DBN S647-phosphorylation of approximately 80% (Figure 5A). Direct inhibition of PI3K did not alter pS647-DBN in neurons (Figure 5B), suggesting that the PTEN induced - PI3K independent - de-phosphorylation of pS647-DBN may be a broad mechanism that operates in different cell types. DBN is highly enriched in the nervous system and present in motile neuronal compartments such as growth cones and dendritic spines [13,35]. We took advantage of the pS647-DBN antibody and analyzed the spatial distributions of this phosphorylation event, and correlated it with the localization of PTEN and DBN. We were particularly interested in the possibility of PTEN induced dephosphorylation of DBN either directly or as part of a protein complex in dendritic spines. In hippocampal neurons (cultured for 1 or 15DIV) the staining pattern of S647 phosphorylated DBN showed particular enrichment in growth cones and in dendritic spines (Figure 5C–F). This was in sharp contrast to the staining pattern of PTEN, which was present in axons and dendrites, but essentially absent in growth cones and dendritic spines. Higher magnification of labeled hippocampal neurons revealed an enrichment of pS647-DBN in dendritic spines, whereas PTEN was mainly present in the dendrite (Figure 5D, E). On occasion, however, PTEN was found in spines (Figure 5E); this coincided with depleted pS647-Drebrin labeling, suggesting a degree of PTEN mobility in entering the spine. To further strengthen a mechanism involving negative regulation of pS647-DBN by PTEN in neurons, we used RFP-CRE expressing *PTEN* ^*flox/flox*^ neurons and performed immunocytochemistry using anti-PS647-DBN. In comparison to control RFP expression neurons, expression of Cre resulted in an increased PS647-DBN labeling. In summary, these results demonstrate that PTEN and pS647-DBN segregate into complimentary compartments, strengthening the idea that PTEN can negatively regulate pS647-DBN in neurons.

### Membrane depolarization induces transient phosphorylation of DBN at S647

**Figure 6:**
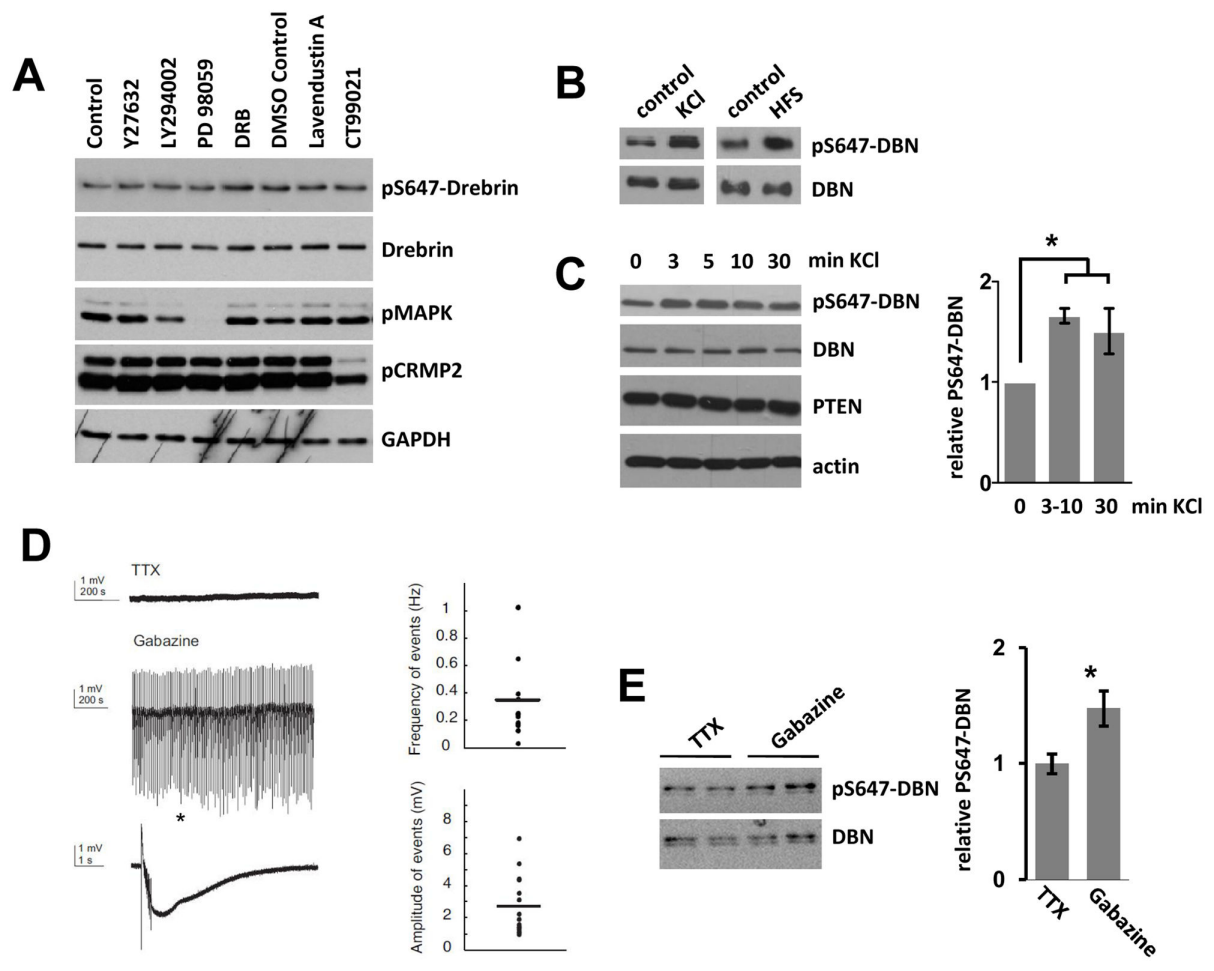
Phosphorylation of DBN at S647-Drebrin is increased by synaptic activity. (A) Cortical neurons were treated with 10 µM Y27632 (Rock inhibitor), 20 µM LY294002 (PI3K inhibitor), 10 µM PD98059 (MAPK inhibitor), 10 µM DRB (CK2 inhibitor), 10 µM Lavendustin A (general kinase inhibitor) or 2 µM CT99021 (GSK-3 inhibitor) and analyzed by western blotting using indicated antibodies. The anti-pMAPK and anti-pCRMP2 blot verified the activity of MAPK and GSK-3 inhibitors, respectively. (B) Hippocampal neurons were treated with KCl (20 mM) for 10 minutes, or with high frequency electrical stimulation (HFS) at 100 Hz, 1 s, twice with a 20 s interval. (C) Neurons were treated with KCl (20 mM) for indicated periods of times. Bar graph represents the average band density of pS647-DBN/DBN at each time point in 3 independent experiments + sem *p<0.05. (D) Organotypic hippocampal slice cultures were exposed to 5 µM gabazine (n = 21) or 500 nM TTX (n = 29) and network activity was recorded for 1 hour in stratum pyramidale of the CA3 subfield. TTX-exposed cultures showed no spontaneous activity whereas gabazine exposed cultures developed seizure-like events. A single event indicated by the asterisk is magnified. Bars demonstrate the frequency (left) of events over a time period of 1000 s and their amplitude (right). (E) Organotypic hippocampal slice cultures were exposed to gabazine or TTX for 1 hour lysed and analysed with the indicated antibodies. Bar graph shows the relative band density of pS647-DBN/DBN in >7 independent samples + sem; *p<0.05.

Spatially restricted control of protein phosphorylation can trigger changes in spine morphology and alters synaptic efficacies. We were interested in analyzing the molecular mechanisms involved in phosphorylating DBN at S647 and thus blocked sequentially major signaling pathways that are known to regulate structural and/or functional plasticity at the synapse using pharmacological inhibitors [36]. None of the pathways investigated (Rock, PI3K, MAPK, CK2, GSK-3) influenced the level of pS647-DBN (Figure 6A). We then tested if neuronal activity alters pS647-DBN, and used KCl treatment or high-frequency electrical stimulation (HFS) to drive synaptic activity in neuronal cultures. Increased network activity resulted in an elevation of pS647-DBN (Figure 6B), which occurred within 3–10 min (Figure 6C). We next asked whether increasing neural network activity also induces pS647-DBN in a more complex preparation. In organotypic hippocampal slice cultures exposed to gabazine, which induces highly synchronized neuronal network activity (seizure-like events) (Figure 6D), we observed a 50% increase in pS647-DBN when compared to slices with TTX to abrogate all spontaneous activity (Figure 6E). These results demonstrate that DBN is phosphorylated at S647 under basal conditions in cultured hippocampal neurons as well as in organotypic hippocampal slice cultures, whilst increased neuronal network activity is able to promote pS647-DBN further in both experimental paradigms.

### PTEN-DBN controls steady-state levels and activity-dependent increases in phospho-S647-DBN

**Figure 7:**
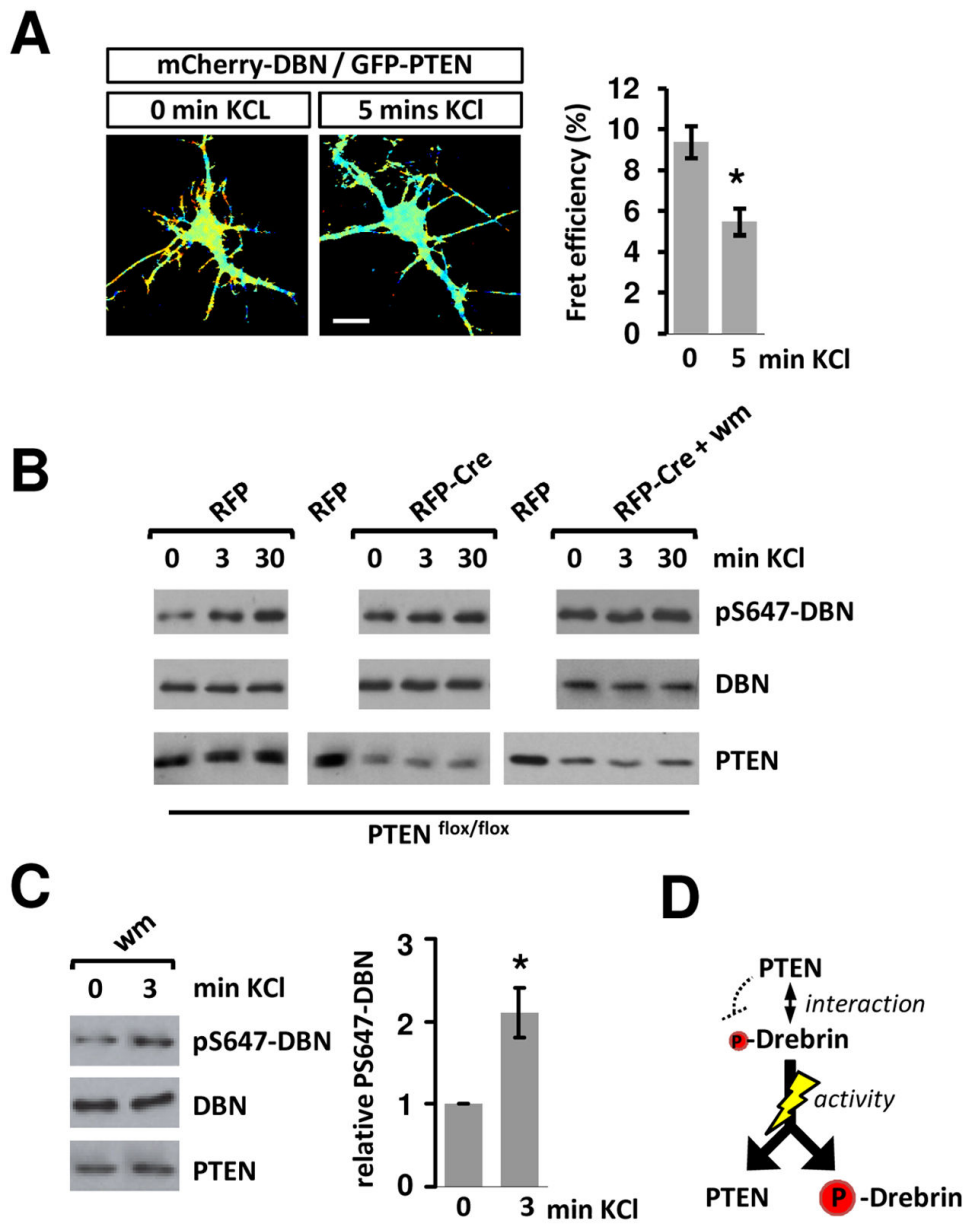
Membrane depolarisation induced dissocation of PTEN-DBN and increases the phosphorylation of pS647-DBN. (A) Neurons were transfected at 7 DIV with mCherry-DBN and GFP-PTEN and incubated until 10 DIV, before treatment with KCl (20 mM) and FLIM measurements. Bar graphs represent the average FRET efficiency of 22 cells over 3 independent experiments + sem. *p<0.001. Scale bar: 10 µm (B) Cortical neurons from floxed PTEN mice expressing RFP or RFP-Cre were cultured for 10 DIV and treated with KCl (20 mM). (C) 11 DIV rat hippocampal neurons were treated with KCl (20 mM) for 3 minutes in the presence or absence of Wortmannin (wm) at 100 nM. Bar graph shows the relative band density of pS647-DBN in three independent experiments +sem; *p<0.05. (D) Schematic diagram illustrating signaling relationships. DBN phosphorylation at S647 is suppressed through physical interactions with PTEN. Synaptic activity induces a transient dissociation of the PTEN:DBN complex and allows maximal DBN-S647 phosphorylation to occur.

We showed that pS647-DBN is negatively regulated through physical interactions with the PTEN phosphatase (Figure 4), which led us to hypothesize that PTEN might be involved in controlling activity-dependent changes in S647 phosphorylation of DBN. In this case, neuronal activity potentially influences the binding of PTEN to DBN. To test this idea, we utilized FLIM measurements in hippocampal neurons expressing GFP-PTEN and mCherry-DBN, and measured GFP-lifetimes following short KCl exposure. Quantification of these experiments revealed a 45% decrease in FRET efficiencies at 5 minutes of KCl membrane depolarization, which is consistent with the idea that membrane depolarization induces a dissociation of the PTEN:DBN complex (Figure 7A). Given that the time-course of the dissociation of PTEN:DBN correlates well with the time-course of the depolarization-induced increases in pS647-DBN, we then asked if the PTEN phosphatase suppresses pS647-DBN in non-stimulated neurons to maintain phosphorylation at dynamic range. Neuronal activity induced dissociation of PTEN:DBN would then release this suppression and allow maximal DBN-phosphorylation to occur. We tested this idea in cortical neurons isolated from *PTEN* ^*flox/flox*^ mice. In comparison to RFP-expressing neurons, PTEN removal through Cre-mediated recombination increased the level of pS647-DBN, as shown previously (Figure 5A). Membrane depolarization induced an increase in pS647-DBN in control neurons, whilst in neuronal populations with decreased PTEN expression, the membrane depolarization-induced increases in pS647-DBN were not observed above elevated levels (Figure 7B). Thus, loss of PTEN was associated with a higher steady-state level of pS647-DBN and prevented any further activity-induced increases in phosphorylation. Under basal control conditions and during membrane-depolarization, the effect of PTEN on pS647-DBN was not affected by wortmannin treatment (Figure 7B,C) highlighting the general independence of PI3K signaling in controlling pS647-DBN.

## Discussion

In this study we identify and characterize the interaction of the PTEN tumor suppressor with DBN, an actin-binding protein highly enriched in dendritic spines. Our results demonstrate that PTEN binds DBN and that this interaction results in the dephosphorylation of a site present in the DBN C-terminus - serine 647. PTEN responds to synaptic activity through dissociation from DBN, leading to a de-repression of phosphorylation of DBN at S647.

Immunocytochemistry analyses reveal that pS647-DBN is present in a neuronal compartment exhibiting high actin turnover, which are largely depleted of PTEN protein. In conjunction with our data demonstrating that PTEN negatively regulates S647-DBN phosphorylation independent of PI3K signaling, this complementary protein distribution suggests that the PTEN phosphatase may target pS647-DBN dephosphorylation, either directly or as part of a higher order protein complex. Direct dephosphorylation of pS647-DBN by PTEN is a feasible molecular mechanism, given that the tumour suppressor can act as a dual-specific protein phosphatase that is able to dephosphorylate tyrosine-, and also - but less efficiently - serine- and threonine-phosphorylated proteins [37]. With regard to serine and threonine-phosphorylated protein substrates, recombinant PTEN was found to show a high degree of specificity for substrates surrounded by extremely acidic amino acids [37], sequences we also recognize as flanking S647. On this basis one can speculate that PTEN may target pS647-DBN directly; though further analysis is required to understand PTEN’s regulatory connection to DBN-S647 phosphorylation fully. With respect to a putative kinase involved in phosphorylating S647-DBN site, the highly negatively charged signature could point to a regulation by CK2. However, inhibition of CK2 by DRB did not alter basal levels in pS647-DBN.

Our data are consistent with the idea that PTEN functions as a suppressor of pS647-DBN and associated cellular responses. Absence of PTEN results in an increased S647-DBN phosphorylation, which approximately reflects levels achieved by activity-induced mechanisms. Consequently, only the presence of the PTEN phosphatase suppressor allows the system to respond with changes in pS647-DBN. Rather than an activity-dependent regulation of a kinase being required for the induction of pS647-DBN, it seems that dissociation of the PTEN-DBN complex may set the degree to which pS647-DBN is changing following membrane depolarization (Figure 7D). The fact that a portion of DBN is readily phosphorylated at S647 under basal conditions suggests that the kinase involved may be dominant over PTEN mediated de-phosphorylation. Alternatively, the decreased abundance of PTEN protein residing deep in the dendritic spine compartment may allow spine associated DBN to maintain high basal levels of pS647-DBN. In fact, the results of our immunofluorescence labeling support the idea that PTEN may exhibit a degree of mobility in the synapses of hippocampal neurons, which, to some extent, is in line with the PTEN localization in growth cones [38].

The role of PI3K/PTEN in dendritic spine formation has been extensively studied. Initial investigations reported that direct PI3K inhibition reduces spine density and that expression of constitutively active PI3K increases the number of filopodia [24]. Other activators of the PI3K pathway, such as insulin or a PI3K-activating transduction peptide, promote dendritic spine formation [39,40]. It is also of note that PTEN functions as an important mediator of spinogenesis in mice by involving a PI3K-mTOR pathway [5,9]. Although DBN modulates spine morphogenesis, and PI3K/PTEN is required for the formation and remodeling of spines, we were not able to reveal a direct link between DBN and this signaling pathway (Figure 3). It is temping to speculate that PTEN could potentially act in a PI3K-dependent fashion to regulate spine morphogenesis, whilst its contributions in PI3K-independent responses may be involved in other neuronal functions. In this context it is of note that loss of Pdk1 (Phosphoinositide-dependent kinase-1), which encodes a positive downstream regulator of the PI3K pathway, appears to be unable to rescue PTEN-mediated deficits in spatial memory [8] and support the idea that PTEN can act independent of PI3K signaling in a context dependent manner.

In summary, we identified a novel mechanism by which PTEN responds to synaptic activity through dissociation from a DBN complex, leading to a de-repression of phosphorylation of DBN at S647. DBN is phosphorylated at numerous additional sites. Finding out whether these sites are phosphorylated simultaneously on the same molecule, function in the same brain region, or exert their functions in coordinated or antagonizing ways will be a key to understanding this important cytoskeletal regulator at the CNS synapse.

## Materials and Methods

### Animal procedures

All animal procedures (breeding/sacrifice) were conducted in accordance with local ethical guidelines and approved animal care protocols (London: KCL Ethics Committee and the Home Office - license 70/6475; Berlin: Institutional Animal Care and Use Committee (IACUC) and the Landesamt für Gesundheit und Soziales (LAGeSo) - license T 0347/11; Heidelberg: Animal Care and Use Committee and the state government of Baden-Württemberg - license T56/11).

### Immunoprecipitations and tandem mass spectrometry

Immunoprecipitation and tandem mass spectrometry were performed as described previously [41]. For co-immunoprecipitation experiments, HEK293 cells were transfected with different constructs using Lipofectamine 2000, lysed in 50 mM Tris pH 7.5; 150 mM NaCl; 1% Triton X-100; 2 µM NaOV and 500 µM NaF and immunoprecipitated with anti-FLAG® M2 Affinity Gel (Sigma) to isolate the 3xFlag-DBN complex. Western blot analysis of immunoprecipitates was performed using anti-FLAG (Sigma), anti-GFP (ABCAM) or anti-PTEN antibodies (Santa Cruz or Cell Signalling).

### Cloning

Full length DBN (accession number NM_004395.3) was amplified by PCR using the primers 5’-gaattcagccggcgtcagcttcagcgg-3’ and 5’-ggatccctaatcaccaccctcgaagccctc-3’ and inserted in p3x-Flag-CMV-7.1 (Sigma). Site directed mutagenesis was performed on parental peYFP-DBN using QuikChange II XL mutagenesis kit (Stratagene). All mutations were validated by sequencing. GFP-PTEN plasmids (accession number NM_000314.4) have been described previously [42]. For FLIM experiments in hippocampal neurons, DBN and PTEN were cloned into pCAX vector. DBN shRNA targeted to a rat/mouse specific sequence gaaccagaaagtgatgtac was constructed into pSilencer 2.0-U6 (Ambion) using the oligonucleotides 5’-gatccgaaccagaaagtgatgtacttcaagagagtacatcactttctggttctcttttttggaaa-3’ and 5’-agcttttccaaaaaagagaaccagaaagtgatgtactctcttgaagtacatcactttctggttcg-3’

### Generation of pS647-DBN antibody

A rabbit polyclonal antiserum was raised against a phospho-serine 647-encoded synthetic DBN peptide (CGYFSQS(P)QEEEF; Eurogentec); S647 corresponds to the sequence of human adult DBN. Antibodies were affinity-purified by Sulfolink column chromatography (Thermo Scientific). Antibodies were eluted from the phospho-peptide column using 100 mm glycine, pH2.5 and dialyzed with PBS. In order to absorb antibodies reacting with non-phosphorylated peptides, the eluate was incubated for 1h with the corresponding non-phosphorylated peptide. Affinity purified DBN phospho-antibodies were stored at -20 °C. In p-peptide inhbition experiments, the phospho-serine 647-encoded synthetic DBN peptide was applied at a concentration of 100 µM.

### Western blot analyses

Western blot analysis and immunocytochemistry were performed using anti-FLAG (Sigma), anti-GFP (ABCAM), anti-DBN-Guinea Pig (Fitzgerald), anti-DBN-mouse M2F6 (Abcam) anti-PTEN (Santa Cruz), anti-Akt (Cell Signalling Technology), anti-pS473-Akt (Cell Signalling Technology), anti GAPDH (Abcam), anti-P44/42-MAPK (Cell Signalling Technology), anti-p514/509-CRMP (kindly provided by Calum Sutherland, Dundee), anti-actin (Millipore) and the generated pS647-DBN (Eurogentec). All antibodies used for western blotting were at 1/1000 except both anti-GFP and anti-actin used at 1/5000 and anti-GAPDH used at 1/30000. In immunohistochemistry applications, the cells were fixed using 4% paraformaldehyde and antibodies were used at 1/200. The drebrin antibody used was anti-DBN (Guinea Pig), unless otherwise stated.

### Cell culture, transfection and viral transduction

HEK293 and U87MG cell lines were purchased from ATCC. All reagents were from Invitrogen unless stated otherwise. Primary rat hippocampal or cortical neurons were prepared from E18 Sprague Dawley rat embryos (Charles River Laboratory). After hippocampi isolation, tissue pieces were dissociated in 0.5 mg/ml trypsin (Worthington) in HBSS for 15 min at 37°C, then washed extensively and gently triturated in Neurobasal medium supplemented with 1% Glutamax, 1% FCS, 2% B27 and 1% Penicillin/Streptomycin using a glass fire-polished Pasteur pipette. Primary mouse hippocampal and cortical neurons were prepared from E15.5 *flox/flox PTEN* mice as described, however, with a further wash in 10% horse serum before trituration. Neurons were plated on glass coverslips (Karl Hecht) previously coated with poly-L-lysine and laminin (both 10µg/ml, Sigma) and maintained in 1% FCS, 2% B27 supplemented Neurobasal medium. Neurons were plated at a density of ~300 cells/mm^2^. All constructs were transfected using Lipofectamine 2000. DNA and lipofectamine were added to Optimem at a ratio of 1µg:1µl and incubated for 30 minutes before transfection. 50% of the medium was removed from the cultures and replaced with fresh medium. The transfection mix was added to the culture medium for 30 minutes before being carefully removed and replaced with a 1:1 ratio of conditioned to fresh medium. HEK293, U87MG and hippocampal neurons were transfected using Lipofectamine 2000 and cortical neurons were transfected using Amaxa Nucleofector kit (Lonza).

For viral transduction, a modified lentiviral vector [43,44] was used, in which a human Synapsin-1 promoter drives the expression of a RFP-Cre transgene. Lentiviruses were produced by co-transfecting HEK293T cells with the lentiviral vector and two helper vectors, pVSVg and pCMV-delta R8.9 [43]. Viral supernatants were collected 48 hrs after transfection, and virus particles were added to cultured floxed PTEN neurons 13 days after plating in ratios ranging from approximately 0.5/1 to 2.5/1, respectively. 6 days post-transduction neurons were harvested in RIPA buffer with freshly added protease and phosphatase inhibitor cocktails (Sigma-Aldrich) and lysates were prepared for Western blotting.

### FRET measurements by Fluorescence Lifetime Imaging Microscopy (FLIM)

Spatial interaction between PTEN and DBN were measured in PC12 cells transfected with different constructs using Lipofectamine 2000. Following fixation, FLIM experiments to measure Förster resonance energy transfer (FRET) and data analysis were performed as described previously [45]. Dissociated E18 hippocampal neurons were transfected at DIV7 with pCAX-mCherry-DBN and/or pCAX-GFP-PTEN and imaged at DIV10.

### Generation of cell-permeate peptides

A plasmid based on pBR322 was constructed incorporating bases coding for the sequence of the D-loop sequence (PGPEETSEKVENGSLCDQEIDSICSIERADN) preceded by a Hisx6 N-terminal tag and the Antennapedia sequence (RQIKIWFQNRRMKWKK). *E. coli* strain BL21(DE3) was transformed with 10ng of plasmid, transferred to 1 L LB broth supplemented with Ampicillin (100µg/mL) and grown overnight at 37°C to mid-log phase (A600 0.5–0.6). Peptides were induced with 1 mM IPTG for 3 h and the bacteria were harvested by centrifugation. Bacterial pellets were treated with Bugbuster Reagent (Novagen, UK) according to manufacturers’ instructions and the lysate was passed through a Ni-NTA His Bind Resin (Novagen). Bound peptide was washed and eluted with imidazole according to manufacturers instructions, and dialyzed overnight. For long term storage, peptides were finally dried and stored at -20^o^C until use.

### Organotypic hippocampal slice cultures and electrophysiological recordings

Six to 8-day-old male Wistar rats were used for the preparation of organotypic hippocampal slice cultures according to the interface method [46,47]. Both preparation and medium exchanges were performed with sterile equipment (HERAsafe, Kendro Laboratory products). Organotypic slices were randomly prepared on a weekly basis from three different animals. After decapitation, the hippocampus was isolated and transversely cut in 400µm-thick-slices with a McIllwain tissue chopper (Mickle Laboratory Engineering Co.Ltd, Surrey, UK). Then the slices were transferred in ice-cold dissection medium (Minimum Essential Medium 1.6% w/v (Gibco, Grand Island, NY, USA) in sterile distilled water, buffered with Tris base 7-9 (Sigma-Aldrich Chemie GbMH, Steinheim, Germany) to pH 7.35). Slices were rigorously bubbled with a gas mixture of 95% oxygen (O_2_) and 5% carbon dioxide (CO_2_) and pair-wise affixed on Millicell^®^-CM, 0.4µm porous membrane inserts (Millipore GmbH, Schwalbach/Ts, Germany). The bottom of each insert was in contact with 1ml of incubation medium (Minimum Essential Medium 1.06% w/v (Gibco, Grand Island, NY, USA), Hanks’ Balanced Salt Solution (Sigma-Aldrich Chemie GmBH) 25% v/v, L-glutamine (Gibco) 2mM, heat-inactivated horse serum (Gibco) 25% v/v, buffered with NaHCO_3_ 58‰ w/v and Trisbase 7-9 (Sigma-Aldrich Chemie GbMH, Steinheim, Germany) to pH 7.35), so that the porous membrane allowed for exchange of nutrients and the gaseous phase for supplementation of oxygen. The slices were incubated at normal air with 5% carbogen dioxide (CO_2_) (UniEquip GmbH, Munich, Germany) at 37°C and the incubation medium was fully exchanged three-times-weekly. All solutions were prepared under the hood and filtered with 0.2 µm pore diameter sterile filters.

For electrophysiological recordings, slice cultures were transferred to a custom-made interface recording chamber (34°C), which was perfused with artificial cerebrospinal fluid (aCSF, NaCl 129mM, NaH_2_PO_4_ * H_2_O 1.25mM, D-glucose 10mM, MgSO_4_ 1.8mM, KCl 3mM, CaCl_2_ * 2 H_2_O 1.6mM, NaHCO_3_ 21mM) at a flow rate of 1.5ml/min, gassed with 95% O_2_ / 5% CO_2_ at 1lt/min. Local field potentials were recorded with silver/silver chloride glass microelectrodes of 3-8 MΩ resistance. Voltage changes were low-pass filtered at 3kHz and digitized at 10kHz using a CED Micro 1401-III® data acquisition unit and Spike2® software (Cambridge Electronic Design Limited). Slice cultures (8-11 DIV) were exposed to either 500nM tetrodotoxin (TTX) (BIOTREND Chemikalien) or 5µM gabazine (SR-95531) (Sigma) in the recording chamber; neuronal network activity was monitored in stratum pyramidale of the CA3 subfield. After recordings, slice cultures were snap frozen and stored at -80^o^C until further processing. For western blot analyses three slices exhibiting no- or seizure-like activity were merged for tissue homogenization. Data analysis was performed in Matlab (Mathworks). The amplitude (in mV) of seizure-like events was calculated from the difference of the maximum to the minimum point, and their frequency (in Hz) from the ratio of occurrences over a time period of 1000s. Data analysis was performed in Matlab (Mathworks, Natick, MA, USA). The amplitude (in mV) of gabazine-induced epileptiform events was calculated from the difference of the maximum to the minimum point, and their frequency (in Hz) from the ratio of occurrences over a time period of 1000s.

### Statistical analysis

Student’s t test was used to analyse differences between two conditions. Quantifications of western blots were analysed against a theoretical mean of 1 using a one-sample test. Variances between three conditions were analysed by two-way analysis of variance (ANOVA) with Bonferroni post test using Prism software.
