# Correction: Phosphorylation of the actin binding protein drebrin at S647 is regulated by neuronal activity and PTEN

**DOI:** 10.1371/journal.pone.0359701

**Published:** 2026-10-12

**Authors:** Patricia Kreis, Rita Hendricusdottir, Louise Kay, Ismini E. Papageorgiou, Michiel van Diepen, Till Mack, Jonny Ryves, Adrian Harwood, Nicholas R. Leslie, Oliver Kann, Maddy Parsons, Britta J. Eickholt

With this Correction, the authors clarify that Fig 1B PTEN panel in [1] is the same as the Fig 1B anti-PTEN panel in [2].

As shown in the underlying blot provided in the Fig. 1b panel of Fig S6 of the Supplementary Information of [2,3], PTEN immunoprecipitation, identified by anti-PTEN western blotting, co-immunoprecipitates Myosin Va as well as a protein labeled “antibody X.” This protein “X” is Drebrin, as reported in [1].

The PTEN panel of Fig 1B reports material from [2], published in 2009 by Springer Nature, which are not offered under a CC BY license and are therefore excluded from this article’s [1] license.

## References

[1] Kreis P, Hendricusdottir R, Kay L, Papageorgiou IE, van Diepen M, Mack T, et al. Phosphorylation of the actin binding protein Drebrin at S647 is regulated by neuronal activity and PTEN. PLoS One. 2013;8(8):e71957. doi: 10.1371/journal.pone.0071957 23940795 PMC3733845

[2] van Diepen MT, Parsons M, Downes CP, Leslie NR, Hindges R, Eickholt BJ. MyosinV controls PTEN function and neuronal cell size. Nat Cell Biol. 2009;11(10):1191–6. doi: 10.1038/ncb1961 19767745 PMC2756284

[3] van Diepen MT, Parsons M, Downes CP, Leslie NR, Hindges R, Eickholt BJ. Erratum: MyosinV controls PTEN function and neuronal cell size. Nat Cell Biol. 2009;11(10):1191–6. doi: 10.1038/ncb1961 19767745 PMC2756284

